# Selectin-Mediated Signaling—Shedding Light on the Regulation of Integrin Activity in Neutrophils

**DOI:** 10.3390/cells11081310

**Published:** 2022-04-12

**Authors:** Anika Cappenberg, Marina Kardell, Alexander Zarbock

**Affiliations:** Department of Anesthesiology, Intensive Care and Pain Medicine, University Hospital Muenster, 48149 Munich, Germany; cappenberg@uni-muenster.de (A.C.); m_kard02@uni-muenster.de (M.K.)

**Keywords:** integrin, selectin, PSGL-1, signaling, shedding, neutrophil, leukocyte recruitment

## Abstract

As a consequence of tissue injury or infection, neutrophils are recruited in a stepwise recruitment process from the bloodstream into the surrounding tissue. Selectins are a family of adhesion molecules comprised of L-, E-, and P-selectin. Differences in expression patterns, protein structure, and ligand binding characteristics mediate distinct functions of each selectin. Interactions of selectins and their counter-receptors mediate the first contact of neutrophils with the endothelium, as well as subsequent neutrophil rolling along the endothelial surface. For efficient neutrophil recruitment, activation of β_2_-integrins on the cell surface is essential. Integrin activation can be elicited via selectin- as well as chemokine-mediated inside-out signaling resulting in integrin conformational changes and clustering. Dysregulation of selectin-induced integrin activation on neutrophils is involved in the development of severe pathological disease conditions including leukocyte adhesion deficiency (LAD) syndromes in humans. Here, we review molecular mechanisms involved in selectin-mediated signaling pathways in neutrophils and their impact on integrin activation, neutrophil recruitment, and inflammatory diseases.

## 1. Introduction

Neutrophils or polymorphonuclear cells (PMNs) are the most abundant circulating leukocytes and an important part of the first line of host defense. PMNs extravasate on a regular basis from the bloodstream into the tissue and watch out for invading pathogens. Additionally, they are able to directionally extravasate in large numbers out of the bloodstream in case of tissue injury or infection. This process occurs in a distinct series of events, which is called leukocyte recruitment cascade [1]. Neutrophil recruitment to sites of infection or tissue injury has to be tightly regulated and balanced to avoid overwhelming inflammation and tissue damage on the one hand, and insufficient immune response leading to severe infections and pathogen spreading all over the body on the other hand [2,3]. The interaction and (de-)activation of different adhesion molecules on the neutrophil surface is important during each step of leukocyte recruitment. The different types of leukocyte adhesion deficiency (LAD) are mediated by dysfunction of distinct adhesion molecules and characterized by recurrent bacterial infections, reflecting the importance of this signaling network [4,5].

Selectins are a family of adhesion molecules, expressed on leukocytes and the inflamed endothelial surface, and are important during the first steps of the leukocyte recruitment cascade, capturing and rolling [6]. Selectin-binding to ligands on the neutrophil surface activates signaling processes resulting in integrin activation. This article will focus on signaling cascades activated in neutrophils downstream of selectin binding to its ligands, the resulting effects on integrin activity as well as the effects of selectin- and integrin-dependent pathways in pathological processes involved in inflammatory diseases.

## 2. Integrins—Big Players during Each Step of Neutrophil Recruitment

Integrins are heterodimeric, transmembrane cell surface adhesion molecules widely expressed on almost all mammalian cell types. As cell surface receptors, integrins are able to mediate cell–cell and cell–extracellular matrix interactions via interaction with their specific ligands. Signal transduction induced by integrin–ligand binding is very important for several immune functions.

Until now, 24 different integrins characterized by the combination of 18 different α- and 8 different β-subunits forming αβ-heterodimers, are described in mammals [7]. Both subunits consist of several domains with flexible linkers and are characterized by a large ectodomain followed by a transmembrane domain and a short intracellular tail. The integrin ectodomains can be subdivided into a headpiece, which mediates ligand binding, and a membrane proximal tailpiece. Conformational changes determine the arrangement of the integrin domains, thereby changing integrin affinity (described in Section 2.2). The intracellular tail is very important for downstream signaling due to its ability to directly bind adapter and signaling proteins [8,9]. The most prominent integrins expressed on leukocytes, β_2_-integrins, always consist of the β_2_-integrin chain (CD18) in combination with a variable α-chain, which is responsible for ligand specificity [10]. On neutrophils, expression of the β_2_-integrins lymphocyte function-associated antigen-1 (LFA-1; α_L_β_2_; CD11a/CD18) and Macrophage antigen-1 (Mac-1; α_M_β_2_; CD11b/CD18) is well-described. Ligands for LFA-1 are intercellular adhesion molecules (ICAMs), mainly expressed on the endothelial surface. Mac-1 is able to bind a variety of ligands including fibrinogen, fibronectin, or complement proteins [11,12]. Further integrins expressed on neutrophils are very late antigen-4 (VLA-4; α_4_β_1_) and small amounts of α_x_β_2_ (gp 150,95) [13].

Several studies demonstrated the importance of LFA-1 and Mac-1 expression on neutrophils, as well as the regulation of their activity by a series of adhesion and signaling molecules, during different steps of neutrophil recruitment from the bloodstream into the surrounding tissue. This will be discussed in detail in the following sections.

### 2.1. Integrins and Their Role within the Leukocyte Recruitment Cascade

**Figure 1:**
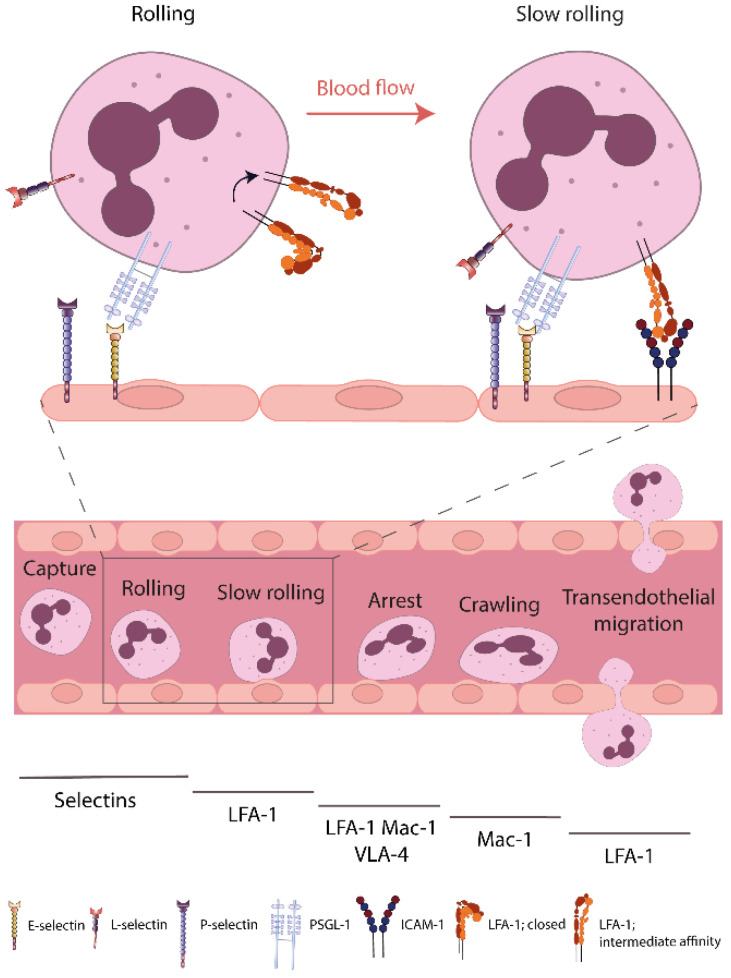
β_2_-integrins during neutrophil recruitment and transition from rolling to slow rolling. In case of tissue injury or infection, neutrophils are able to directionally extravasate in large numbers out of the bloodstream into the tissue. Activity regulation of β_2_-integrins on the neutrophil surface is very important for different steps of the leukocyte recruitment cascade. Selectin-dependent integrin activation during neutrophil capturing and rolling mediates the activation of LFA-1, necessary for neutrophil slow rolling and subsequent recruitment. Key molecules involved in different recruitment steps are indicated below. ICAM-1, intercellular adhesion molecule-1; LFA-1, lymphocyte-function-associated antigen-1; Mac-1, macrophage antigen-1; PSGL-1, P-selectin glycoprotein ligand-1; VLA-4, very late antigen-4.

Under resting conditions, immune cells including PMNs circulate within the bloodstream. However, in case of tissue injury or infection, inflammatory stimuli are recognized by immune cells and a directional extravasation out of the vessel into the surrounding tissue takes place via the leukocyte recruitment cascade [1]. Multiple steps of the cascade are dependent on adhesion molecules, especially selectins and integrins, expressed on the leukocyte as well as endothelial surface (Figure 1). Via receptor–ligand interactions as well as signal transduction into the cell, leukocyte recruitment is tightly regulated [14].

First of all, leukocytes have to contact the inflamed endothelium. This process is called capturing [15,16], and the subsequent rolling along the vessel wall is mediated by interaction of selectins with their respective ligands [17,18]. A detailed description of the selectin adhesion molecule family and their role during leukocyte recruitment and integrin activity regulation will follow in Section 3. The subsequent recruitment steps are dependent on expression and function of distinct integrins on the neutrophil surface. Rolling neutrophils decrease their velocity via binding of activated β_2_-integrins to their ligands on the endothelial surface. Several studies demonstrated that the binding of LFA-1 on neutrophils to ICAM-1 on the inflamed endothelium is necessary for slow rolling [19,20,21]. Furthermore, neutrophil activation during slow rolling via interaction with inflammatory stimuli, including cytokines and chemokines presented on the endothelium, causes stronger neutrophil–endothelial interaction resulting in arrest and adhesion strengthening [1,22]. These processes are dependent on LFA-1 binding to ICAM-1, but VLA-4 and Mac-1 are also involved. Following adhesion, neutrophils crawl along the endothelial cell layer, searching for a specific site to transmigrate into the tissue. Neutrophil crawling is dependent on Mac-1 function and the ligation of ICAM-1 [23,24]. Neutrophil transendothelial migration is possible via a transcellular or a paracellular route and is also highly dependent on β_2_-integrins. This complex process is described in detail in several reviews and is not the focus of this article [25,26]. More recent investigations identified a mechanism referred to as reverse transmigration. During reverse transmigration, neutrophils that were already migrated outside the vessel migrate back into the vasculature [27,28,29]. This mechanism is predicted to be a part of the proper resolution of an inflammatory reaction. Further mechanisms are involved in regulation of the inflammatory response. The expression of a series of signaling and adhesion molecules, including selectins and integrins, is regulated underlying a circadian rhythm, thereby also affecting leukocyte recruitment [30,31].

### 2.2. Integrin Characteristic Trait: Conformational Changes and Clustering

Integrins are able to transmit signals bidirectionally through the plasma membrane via pathways named inside-out and outside-in signaling. In the context of integrin activity and function, three terms are important to know: integrin affinity, valency, and avidity [1]. Integrin affinity is dependent on integrin heterodimer conformation and characterized by distinct ligand binding capacities and dissociation rates. Integrin valency describes a more spatial integrin characteristic. Due to lateral mobility and variable expression levels, the density of integrin molecules on distinct areas of the cell surface can differ (clustering) and, thus, be responsible for altered cell adhesion. Integrin clustering probably allows rapid responses to integrin ligands, also in lower concentrations. The combination of integrin affinity and valency determines a more macroscopic determinant for integrin adhesiveness: avidity.

Inside-out signaling can be induced by various stimuli and results in increased integrin–ligand binding affinity, up to a ~10,000-fold increase [32]. Via induction of conformational changes, ectodomain extension is promoted, resulting in opening of the ligand binding pocket [33]. Extension of the integrin ectodomain also facilitates clustering. Integrin avidity modulation is essential for different steps of the leukocyte recruitment cascade through regulation of integrin–ligand interactions, thereby controlling neutrophil–endothelial interactions. β_2_-integrins can appear in at least three different conformations [7,34,35]. On circulating resting neutrophils, integrins are mainly present in a nonadhesive state with bent ectodomain and a closed headpiece (E^−^H^−^) representing a very low ligand binding capacity. Depending on the inflammatory signal, the integrin conformation changes into an extended conformation resulting in integrin activation and clustering. During activation, integrin cytoplasmic tails separate, causing unfolding of the integrin ectodomain [7]. The extended ectodomain with a closed headpiece (E^+^H^−^) exhibits intermediate ligand binding affinity [36,37]. The full open conformation with extended ectodomain and open headpiece (E^+^H^+^) is characterized by high ligand binding affinity. More conformations are discussed in recent publications [38,39]; however, these are not relevant for the topic of this review.

In contrast to inside-out signaling, integrin outside-in signaling describes signaling processes inside the cell downstream of ligand-induced integrin clustering. During inflammatory processes, outside-in signaling mostly occurs in parallel with other signaling events, making it difficult to have an isolated view on its signaling function. Via binding to extracellular matrix proteins, integrin clustering can be induced via outside-in signaling. Cell behavior including adhesion, cytoskeletal reorganization and cell spreading can be affected as a consequence of changes in the inflammatory milieu [40]. In terms of neutrophils, integrin outside-in signaling is important for neutrophil effector functions including degranulation, production of reactive oxygen species, and phagocytosis [11].

## 3. Selectins and PSGL-1: The Underestimated Basis for Neutrophil–Endothelial Interactions

**Figure 2:**
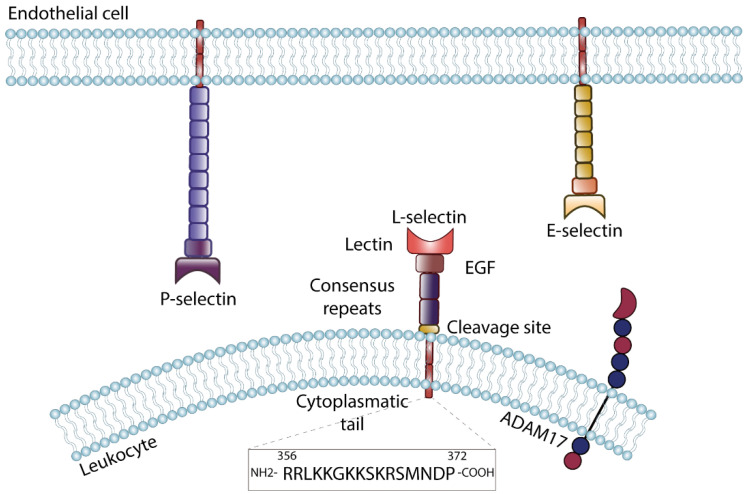
Selectin structure and expression. The selectin family consists of three members: E-selectin is expressed on the surface of activated endothelial cells, P-selectin occurs on endothelial cells as well as on platelets. L-selectin is expressed on leukocytes and can be shed by specific enzymes including A disintegrin and metalloproteinase 17 (ADAM17). Selectins share a common structure, characterized by an N-terminal C-type lectin domain, followed by an epidermal-growth-factor-like domain (EGF) and a number of consensus repeats, which is characteristic for each selectin. The transmembrane domain is followed by a short cytoplasmic tail. The cytoplasmic tail of L-selectin (amino acid sequence shown at the bottom) is highly conserved between species and is able to fulfill signaling function.

Selectins are adhesion molecules represented by three family members; L-, P-, and E-selectin. The type I transmembrane glycoproteins mediate interaction between hematopoietic cells and to the endothelial surface [41]. All three selectin genes are arranged in tandem on (human and murine) chromosome 1, suggesting a duplication of one originating gene during evolution [42]. Selectins are structurally characterized by an N-terminal C-type lectin domain, followed by an epidermal-growth-factor-like domain, a series of consensus repeats, and a transmembrane domain with a short intracellular tail [43] (Figure 2). The number of consensus repeats is specific for each selectin, L-selectin is the smallest selectin with only two consensus repeats, E-selectin is characterized by six and P-selectin by nine consensus repeats. Due to the differences in size, ligand binding capacities of the selectins are different. Although the general structure is shared, the expression pattern of each selectin is quite unique. L-selectin (CD62L) is ubiquitously expressed on leukocytes and some stimuli induce proteolytic cleavage of the L-selectin ectodomain [44]. In contrast, E- (CD62E) and P-selectin (CD62P) are expressed on the surface of endothelial cells and P-selectin also on platelets. E- and P-selectin expression is absent in resting endothelial cells, but is upregulated following exposure of the endothelium to proinflammatory stimuli such as tumor necrosis factor (TNF)-α, interleukins, or lipopolysaccharide [41]. Upon stimulation, P-selectin is mobilized from Weibel–Palade bodies in endothelial cells as well as α granules in platelets and transported to the cell surface on short call in case of inflammatory signals [45]. E-selectin is regulated on the transcriptional level and its availability on the cell surface is therefore delayed in time [41].

Selectins mediate capturing of neutrophils to the endothelium, or interaction among neutrophils, via calcium-dependent binding of the lectin domain to carbohydrate moieties on glycoprotein ligands. The minimal structural determinant required for a selectin ligand is a branched tetrasaccharide named sialyl Lewis x (sLe^x^; Siaα2,3Galβ1,4[Fucα1,3]GlcNAc) [46]. Several publications demonstrated that the expression of 2 α1,3-fucosyltransferases are required for selectin-dependent leukocyte rolling on the endothelial surface [47,48]. Different selectin ligands such as ESL-1 [18], Mac-1 [49], and CD44 [50] are known so far; however, the best-described selectin ligand on the leukocyte surface is P-selectin glycoprotein ligand-1 (PSGL-1) [51]. PSGL-1 is a type I transmembrane disulfide-linked homodimeric sialomucin, which is able to bind E- [51], P- [52], and L-selectin [16]. As PSGL-1 is located in lipid rafts on top of leukocyte microvilli [53], it is easily accessible for ligand binding. Studies using blocking antibodies suggest a common or at least overlapping binding site for L- and P-selectin at the NH_2_-terminal region [16,52,54]. In contrast, E-selectin is still able to bind PSGL-1 in these studies, indicating a different or additional E-selectin binding site on PSGL-1. The big extracellular domain of PSGL-1 is rich in threonines, prolines, and serines and is characterized by a series of 14–16 decameric repeats and an NH_2_-terminal signaling peptide. The transmembrane domain is followed by a small intracellular domain. For selectin-binding ability, several posttranslational modifications of PSGL-1 are necessary. The NH_2_-terminal domain of PSGL-1 carries three tyrosine residues, and at least one of them has to be sulfated by tyrosyl sulfotransferases for effective binding of P-selectin [55,56]. Different glycosyltransferases are described to be responsible for modifications of the threonine residue next to the tyrosine sulfate motif, which are necessary posttranslational modifications for the interaction with P- as well as L-selectin. Protein O-glycosylation of serine and threonine residues via polypeptide N-acetylgalactosamine transferase (ppGalNAcT) is also necessary for proper selectin binding. Neutrophils deficient for ppGalNAcT-1 demonstrate a defective rolling behavior in vitro [57] as well as in vivo [58]. The transmembrane and cytoplasmic domains of PSGL-1 are highly conserved. The cytoplasmic domain interacts with several intracellular adapter and signaling proteins, thereby transducing signals into the cell after ligand binding. This quality is very important for the signaling pathways described in the next chapter.

During leukocyte rolling on the endothelium, rapid but reversible bonds between selectins and selectin ligands have to be formed to mediate stable rolling also at high shear stresses. Neutrophil rolling has been investigated extensively in postcapillary venules in vivo and flow chamber assays in vitro at different shear stress levels [59,60]. Rapid formation, but also dissociation of selectin–ligand interactions, are needed to promote rolling. Selectin–ligand interaction becomes stronger with increasing pulling forces until a certain cutoff, a phenomenon called catch-bond [61,62]. In the second phase, at high pulling forces, selectin–ligand bonds show slip bond characteristics with higher dissociation rates [61,63]. This catch–slip transition promotes rapid bond formation at the front of the rolling cell and stabilizes the bond during rolling over the cell center. Rapid dissociation at the cell rear, allowing further rolling along the endothelium, is promoted after transition into the slip-bond phase. Catch bond formation of human E-selectin with sLe^x^ on neutrophil L-selectin is also able to mechanotransduce intracellular signaling, resulting in conformational changes into the integrin high-affinity state and subsequent neutrophil arrest [64,65,66].

During neutrophil rolling on the inflamed endothelium, several mechanisms to stabilize rolling are described. PSGL-1 is located on microvilli on the cell surface. During tethering and rolling, a high force is exerted on the PSGL-1–selectin bond mediated by blood flow shear stress. Due to the forces, microvilli can extend via detachment of the plasma membrane from the cytoskeleton and form cellular protrusions called ‘tethers’ [67,68]. When tether anchoring points break, the membrane protrusion can wrap around the rolling neutrophil, forming a so-called ‘sling’. Approximately 15% of neutrophil tethers become slings after detachment. Tethers and slings stabilize rolling by increasing the cellular surface and the number of interaction points for the neutrophil [68,69].

### 3.1. Selectin-Ligand Engagement Induces Inside-Out Signaling in Neutrophils

During leukocyte recruitment, selectins are important in order to mediate the first contact between leukocytes and the inflamed endothelium. Each selectin has distinct characteristics in ligand-binding, ligand-dissociation, and the elicited downstream signaling, resulting in different functions. In this section, we will describe in detail the commonalities but also important differences in downstream signaling following selectin–ligand binding and the effects on leukocyte recruitment.

**Figure 3:**
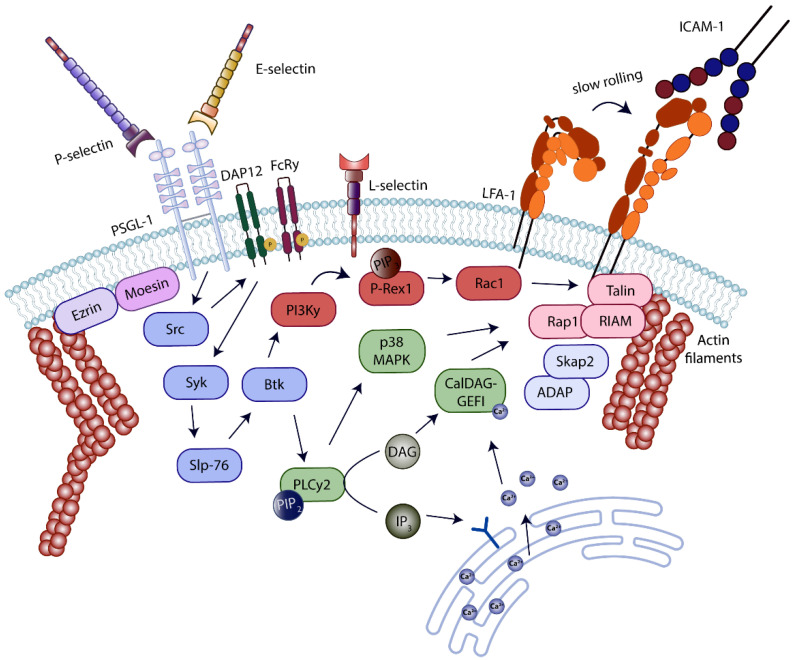
Inside-out signaling cascade in neutrophils induced by selectin–ligand binding. Selectin-binding to P-selectin glycoprotein ligand-1 (PSGL-1) on the neutrophil surface, elicits a specific intracellular signaling pathway resulting in affinity activation of lymphocyte-function-associated antigen-1 (LFA-1). Downstream of selectin-PSGL-1-ligation, several signaling molecules including kinases, adapter proteins, and GTPases are activated. The complex signaling machinery results in conformational changes of LFA-1 from a bent conformation with low ligand binding affinity to an extended conformation with intermediate ligand binding affinity. Extended LFA-1 is able to bind ICAM-1 on the endothelial surface, promoting the transition from neutrophil rolling to slow rolling.

First assumptions that E-selectin is involved in β_2_-integrin activation came up in 1991, when Lo and colleagues reported that neutrophils adhering on TNFα-stimulated human umbilical vein endothelial cells (HUVECs) presented activated Mac-1 [70]. Signaling events downstream of E-selectin binding to PSGL-1 on the surface of neutrophils, taking place during neutrophil rolling along the endothelium under inflammatory conditions, were investigated extensively within the last 15 years. Many publications uncovered a complex signaling network resulting in a partial activation of the β_2_-integrin LFA-1, enabling ligand binding and a decrease in neutrophil rolling velocity (Figure 3).

Successful signaling and integrin activation require intact lipid rafts on the plasma membrane as well as the cytoplasmic domain of PSGL-1 [71]. Following E-selectin binding to PSGL-1, the three Src kinases Fgr, Hck, and Lyn are phosphorylated [72]. Fgr is indispensable for neutrophil slow rolling in vitro as well as in vivo [73]. Downstream of Src kinase activation, the immunoreceptor tyrosine-based activation motif (ITAM)-containing adaptor molecules DAP12 and FcRγ become phosphorylated. Acting as linker proteins, they associate with spleen tyrosine kinase (Syk) [73]. DAP12 and Syk phosphorylation is affected in neutrophils deficient for Fgr or Hck and Lyn [71,73]. Another study reports an interaction of the cytoplasmic PSGL-1 domain with the ITAM-containing adaptor molecules Ezrin and Moesin and a subsequent interaction with and activation of Syk in HL-60 cells [74]. Ezrin and Moesin are also expressed in primary neutrophils; however, the deletion of both DAP12 and FcRγ, results in abolished E-selectin-mediated signaling and neutrophil slow rolling. These data indicate that Ezrin and Moesin and DAP12 and FcRγ are responsible for different cellular responses downstream of PSGL-1 engagement. Ezrin/radixin/moesin (ERM) proteins interact with actin filaments, thereby linking PSGL-1 to the cytoskeleton. ERM proteins as well as PSGL-1 move to the uropod of migrating cells upon polarization, indicating that their interaction is important during later steps of leukocyte recruitment [75].

Further downstream in the signaling pathway induced by E-selectin PSGL-1 ligation, the Tec family kinase Bruton’s tyrosine kinase (Btk) gets phosphorylated and activated in a Syk-dependent manner [71,76]. The immune-cell adaptor SH2 domain-containing leukocyte phosphoprotein of 76 kDa (Slp-76) is required for proper Btk activation [77]. Slp-76 is an immune cell adaptor molecule carrying three distinct tyrosine residues in its N-terminal acidic domain, tyrosines 112, 128, and 145. Via phosphorylation of these tyrosines, proteins can be recruited to a Slp-76 signaling complex. Different studies demonstrated the requirement for Slp-76 expression for E-selectin-dependent Btk activation; however, the studies report different results in structure–function analysis [77,78]. Btk activity initiates two parallel pathways, which cooperatively result in LFA-1 activation and are involved in E-selectin-dependent slow leukocyte rolling in vitro and in vivo. One branch of these parallel pathways is dependent on phospholipase C (PLC) γ2 and the other one on phosphoinositide 3-kinase (PI3K) γ [76]. Blocking one of the two parallel pathways partially abrogates E-selectin-mediated slow rolling, but elimination of both PI3Kγ as well as PLCγ2 activity completely abolishes E-selectin-dependent slow rolling in vitro [76]. Another study by Yago and colleagues suggested that downstream of E-selectin binding to CD44, a common signaling pathway to the E-selectin–PSGL-1 pathway, is elicited, since blocking SFKs, Syk, or Tec kinases abolished slow neutrophil rolling in PSGL-1-deficient neutrophils. Both glycoproteins can compensate the absence of the other. However, this study could not confirm a contribution of PI3Kγ for E-selectin-dependent LFA-1 activation in neutrophils [71]. Most likely, differences in experimental conditions may be responsible for the diverging results. Since neutrophil rolling is very sensitive and can be affected by a series of experimental conditions, isolation of neutrophils from whole blood activates the cells and the appearance of different surface markers is influenced. Additionally, selectin-binding to its ligands is extremely dependent on the shear flow rate, thereby influencing neutrophil rolling behavior [59,79]. Both studies used intravital microscopy of the inflamed murine cremaster muscle as well as flow chamber experiments to identify the involvement of signaling molecules in the process of neutrophil slow rolling; however, the experimental conditions are characterized by huge discrepancies. The intermediate phenotype in rolling velocities of PI3Kγ-deficient neutrophils is pretty small, though this difference is probably not detected in more unphysiological experimental setups.

PLCγ2, regulating one of the two parallel pathways required for LFA-1 activation downstream of E-selectin-PSGL-1 ligation, catalyzes the conversion of phosphatidylinositol,4,5-biphosphate (PIP_2_) into diacylglycerol (DAG) and inositol-3,4,5-triphosphate (IP_3_) [76]. IP_3_ subsequently induces a signaling pathway resulting in an increased intracellular calcium concentration, via mobilization of Ca^2+^ from nonmitochondrial stores. Intracellular signaling molecules of the CalDAG-GEF/RasGRP family are known to provide binding sites for Ca^2+^ as well as DAG. CalDAG-GEFI is expressed in megakaryocytes, platelets, and neutrophils and plays an important role in the distal signaling pathway linking PLCγ2 to E-selectin-mediated LFA-1 activation in neutrophils [80]. E-selectin-dependent neutrophil slow rolling in vitro as well as in vivo is impaired in neutrophils lacking CalDAG-GEFI [80]. The guanine nucleotide exchange factor (GEF) domain of CalDAG-GEF family members is important for the activation of Ras-related protein 1 (Rap1) GTPases. Many studies demonstrate an involvement of Rap1 during GPCR-induced signaling in neutrophils [81,82]. An involvement of Rap1a in E-selectin-dependent neutrophil slow rolling was demonstrated in 2011 by using dominant negative, constitutive-active, or wildtype Rap1a TAT-peptides. In addition, Rap1a is activated downstream of PLCγ2 in a CalDAG-GEFI-dependent manner following stimulation of isolated PMNs with E-selectin in vitro. Following E-selectin engagement, p38 mitogen-activated protein kinase (p38 MAPK) is phosphorylated and involved in neutrophil recruitment steps [73]. The study of Stadtmann and colleagues demonstrated the involvement of p38 MAPK in the PLCγ2-CalDAG-GEFI-dependent part of the signaling pathway resulting in LFA-1 activation [80]. Thereby, PLCγ2 is upstream of CalDAG-GEFI and p38 MAPK, and both molecules are involved in Rap1a activation.

The parallel signaling branch, required for LFA-1 activation, is dependent on PI3Kγ [76]. PI3Kγ activity increases phosphatidylinositol (3-5)-trisphosphate (PIP_3_) production, which is a second messenger that may activate the guanine exchange factor P-Rex1, one of a series of different GEFs able to activate the small GTPase Ras-related C3 botulinumtoxin substrate (Rac) 1 [83]. As a consequence, E-selectin-dependent slow neutrophil rolling is affected in P-Rex1-deficient mice as well as after blocking Rac1 activity, comparable to the phenotype observed in PI3Kγ^−/−^ mice [23].

FERM domain-containing proteins are well-studied in the process of integrin activation and leukocyte recruitment, due to their role in the occurrence of human immune deficiency diseases. Mutations of the FERMT3 gene encoding kindlin-3 are known to be the source for leukocyte adhesion deficiency III (described in detail in Section 4) [84]. Members of the talin and kindlin protein families are most likely involved in the affinity regulation of almost all integrins [85]. Kindlin-3 as well as talin-1, another FERM domain-containing cytoskeletal protein expressed in leukocytes, bind to specific binding motifs in the integrin cytoplasmic tail and are required for integrin activation and adhesive functions. Binding of talin-1 and kindlin-3 to the integrin cytoplasmic tail stabilizes the thermodynamically unfavorable extended β_2_-integrin conformation [85]. In 2012, the individual functions of talin-1 and kindlin-3 in mediating β_2_-integrin affinity conformation changes on neutrophils downstream of distinct stimuli were revealed [21]. Downstream of E-selectin–PSGL-1 ligation, talin-1 binding to the cytoplasmic tail of LFA-1 is essential for LFA-1 extension from the closed to the intermediate affinity conformation in murine and human cells. Neutrophil slow rolling is dependent on talin-1 expression under isolated ex vivo conditions, as well as in a more complex in vivo model [21]. Rap1a is activated upstream of talin-1 in this signaling cascade. The same study demonstrated that the binding of both FERM domain-containing cytoskeletal proteins, talin-1 and kindlin-3, is required for neutrophil arrest and conformational changes of LFA-1 into the high-affinity conformation following chemokine ligation and G-protein-coupled receptor signaling. The recruitment of talins and kindlins to the plasma membrane, enabling binding to the integrin cytoplasmic tail, requires further signaling events. Talin-1 C-terminal domain is able to interact with the actin cytoskeleton on the one hand, but also binds many different signaling and adaptor molecules on the other hand. Rap1 interacting adaptor molecule (RIAM) is able to bind talin-1 and Rap1 simultaneously, thereby forming a complex necessary for talin-1 membrane recruitment during β_2_-integrin activation [86,87]. In addition, another cytosolic adapter protein, the Src kinase-associated phosphoprotein 2 (Skap2), expressed in a variety of cell types including hematopoietic cells, is described to be a binding partner of RIAM in activated leukocytes. Skap2 is activated by PIP_3_ binding to its PH domain and is required for tyrosine phosphorylation of the adhesion and degranulation-promoting adapter protein (ADAP), prior mediating Rap1-RIAM-talin-1 complex recruitment to the plasma membrane [88,89]. In addition to ADAP and RIAM binding, Skap2 was described in neutrophils to interact with Wiskott–Aldrich syndrome protein (WASP) downstream of E-selectin–PSGL-1 ligation. This interaction is indispensable for β_2_-integrin activation and E-selectin-dependent neutrophil slow rolling [90]. Several studies indicate that the adhesion and signaling complex formed at the integrin cytoplasmic tail during integrin activation is formed by a high number of molecules. For example, a recent publication by Vadillo and colleagues demonstrated a role of the actin-binding motor protein Myosin1e in LFA-1 activation mediated via neutrophil rolling on the inflamed endothelium [91]. Further experiments are needed to fully elucidate the underlying signaling pathways responsible for the observed phenotype in neutrophil rolling, described as ‘intermittent rolling’. Another work reports a role of the metalloproteinase ADAM8 in complex with Myosin1f in neutrophil rolling and recruitment under inflammatory conditions, supporting the involvement of Myosin proteins in integrin activation during neutrophil rolling and interaction with the inflamed endothelium [92].

Neutrophil rolling on P-selectin and P-selectin binding to PSGL-1 can also trigger LFA-1 activation [93]. Kuwano and colleagues demonstrated that the signaling cascades downstream of PSGL-1 ligation by either E-selectin or P-selectin are overlapping in many signaling molecules including Fgr, Syk, PLC, and p38 MAPK [20].

Most studies describing selectin-dependent integrin activation, focus on approaches that are designed to mechanistically identify signaling outcomes on changes in integrin affinity. However, integrin avidity changes are also dependent on integrin clustering. Some regulators of integrin conformational changes also affect β_2_-integrin clustering on the neutrophil surface [90,94]; however, the detailed mechanisms have to be further investigated. In contrast, there are additional reports indicating signaling molecules exclusively involved in mediating either integrin clustering or affinity changes on leukocytes [95].

### 3.2. L-Selectin and Its Special Role during Integrin Activation and Leukocyte Recruitment

In contrast to the other selectins, L-selectin is expressed on most circulating leukocytes. In consideration of its size, glycosylation pattern, and functions, L-selectin differs in contrast to the other members of the selectin adhesion molecule family. Within the leukocyte recruitment cascade, L-selectin is involved in initiation of leukocyte tethering to and subsequent rolling on inflamed endothelial surface. However, there is a large body of evidence suggesting an important function of L-selectin-dependent signaling for neutrophil adhesion and migration [96,97,98]. To increase tethering efficiency, L-selectin is located on microvilli on the leukocyte surface. The location on these fingerlike protrusions facilitates the L-selectin–ligand interaction [99]. In neutrophils, L-selectin regulates trafficking to inflamed tissues; however, the exact intracellular signaling pathways have to be further investigated in detail. On human neutrophils, L-selectin itself is decorated with sLe^x^, the minimal structural determinant required for a selectin ligand, and is thereby able to serve as a ligand for E-selectin [64,100]. Ectodomain shedding from the cell surface at a specific membrane-proximal cleavage site via matrix-metalloproteinases (MMP) is another characteristic of L-selectin [101]. Several functions of shedding during inflammatory processes are described within the literature; additionally, L-selectin shedding is used to assess cellular activation in vivo and in vitro. Loss of L-selectin on the cell surface is inversely correlated with Mac-1 expression in activated neutrophils [102]. The released extracellular L-selectin domain (sL-selectin) represents a soluble bioactive ligand within the circulation [103]. The serum sL-selectin level is determined as a readout in several inflammatory diseases. In vitro studies suggested a role of L-selectin shedding for the efficiency of neutrophil transendothelial migration; however, the mechanisms involved remain elusive [104,105]. In terms of β_2_-integrin-dependent signaling in neutrophils, a recent publication demonstrated an important role of L-selectin shedding [106], which was demonstrated to be crucial for neutrophil recruitment and bacterial clearance in the lung. Integrin-mediated outside-in signaling was amplified by L-selectin shedding, resulting in an increased neutrophil migration capacity, production of reactive oxygen species, and level of microbial phagocytosis [106]. A disintegrin and metalloproteinase 17 (ADAM17; or tumor necrosis factor-alpha-converting enzyme (TACE)) is the main enzyme responsible for L-selectin shedding in leukocytes in cis (Figure 2) [107]. Mechanistically, ADAM17 is activated in an IRhom2-dependent pathway downstream of β_2_-integrin ligation on neutrophils [106]. A recent study by Singhal and colleagues confirmed the role of L-selectin shedding during neutrophil recruitment into the inflamed lung, and additionally suggests a Rho-dependent pathway responsible for shedding induction [108]. The Ivetic lab demonstrated in 2021 for the first time a coclustering of L-selectin with platelet-endothelial cell adhesion molecule-1 (PECAM-1) during neutrophil transendothelial migration [109]. L-selectin shedding is intensified via this mechanism resulting in faster transendothelial migration in vitro. This observation was specific for TNFα-activated monolayers. L-selectin shedding is also described to regulate neutrophil rolling in murine cells; however, this observation could not be confirmed in the human system [104,110].

The short intracellular tail of L-selectin, only comprising 17 amino acids, has distinct functions for the transduction of inflammatory signals into the cell. It is able to directly bind intracellular proteins, including the ERM proteins α-actinin and calmodulin [96,111]. In the nineties, several publications demonstrated that clustering of L-selectin either with monoclonal antibodies or physiological ligands induced activation of intracellular signaling molecules including MAP kinases [112], but also resulted in activation of β_1_- and β_2_-integrins [113,114]. In neutrophils, L-selectin can cocluster with a subset of PSGL-1 molecules in cis. This PSGL-1-L-selectin complex formation is required for triggering inside-out signaling resulting in LFA-1 activation [72]. E- or P-selectin, expressed on the inflamed endothelium bind to the PSGL-1–L-selectin complex. The same study demonstrated an (indirect) binding of the intracellular tails of L-selectin and PSGL-1 to the Src kinases Fgr, Hck, and Lyn, which is indispensable for selectin-dependent integrin activation [72].

### 3.3. Integrin-Deactivation: Important Mechanisms to Keep the Balance

All signaling pathways and mechanisms described in previous paragraphs promote neutrophil and integrin activation. However, inflammatory reactions have to be tightly regulated and adequate control mechanisms have to be integrated to prevent overwhelming immune responses resulting in tissue destruction. Further, for efficient leukocyte migration and recruitment, integrin activation and subsequent deactivation is required. Uncovering signaling pathways limiting inflammatory signals may also provide new approaches for the generation of anti-inflammatory drugs.

One endogenous, well-known anti-inflammatory mechanism is promoted via anti-inflammatory cytokines such as IL-6, IL-10, and TGF-β [115]. In terms of β_2_-integrin regulation, several signaling molecules including phosphatases, growth factors, and GTPase activating proteins (GAPs) were identified to have inhibitory effects on integrin activity [82,116]. A prominent negative regulator of inflammatory reactions is the tyrosine phosphatase Src homology 2 domain-containing protein tyrosine phosphatase 1 (Shp-1), which is expressed in all hematopoietic cells [117]. The so-called motheaten mouse phenotype is mediated by a homozygous mutation of the gene *Ptpn6*, which encodes Shp1. The motheaten phenotype is characterized by severe chronic skin inflammation and production of auto-antibodies [118]. After transplantation of motheaten donor bone marrow into wildtype recipient mice, a LFA-1-dependent inflammatory phenotype develops [119]. In neutrophils, Shp-1 negatively regulates selectin- and chemokine-induced β_2_-integrin activation and neutrophil recruitment. Mechanistically, Shp1 phosphatase activity is decreased downstream of selectin- or chemokine-binding via phosphorylation of the serine in position 591. Shp1 interacts with the kinase PIPKIy in neutrophils, thereby regulating PtdIns(4,5)P_2_ levels. In stimulated Shp1-deficient neutrophils, PtdIns(4,5)P_2_ levels are significantly elevated, resulting in integrin overactivation [94].

## 4. The Crucial Role of Selectins and Integrins in the Development of Human Diseases

Over the past decades, leukocytes, particularly neutrophils, have been shown to play a crucial role in pathological inflammation and many different clinical disease models including bacterial pneumonia [120], cecal ligation puncture (CLP) [121], ischemia-reperfusion-injury (IRI) [77], and experimental autoimmune encephalomyelitis (EAE) [122]. Adhesion molecules are the main contributors to regulate neutrophil recruitment into damaged tissue. Among the different recruitment steps, selectin–ligand binding and integrin activity regulation are of particular importance. A defect or dysfunction during leukocyte recruitment leads to inflammatory diseases including immunodeficiency syndromes and recurrent infections.

The leukocyte adhesion deficiency (LAD) syndromes, first described by Anderson and Springer in 1987, are the most-known congenital diseases when it comes to dysfunctions of selectins and integrins in leukocyte recruitment [123]. The hereditary leukocyte adhesion and migration defect results in severe bacterial infections in human patients. To date, three types of LAD have been well-described [4]. LAD I is defined as autosomal recessive disorder caused by mutations in the ITGB2 gene encoding the CD18 chain of β_2_-integrins. Due to the absence or profoundly reduced expression of β_2_-integrins, migration events are prevented; thus, adhesion and transmigration are severely impaired [124]. Clinically, LAD I is characterized amongst other things by delayed separation of the umbilical cord, recurrent staphylococcal and gram-negative bacterial infections, and perirectal abscesses [125,126]. In LAD I patients, leukocytosis is frequently observed, accompanied with an increased neutrophil count, up to 20 times more compared with the normal white blood cell count [124]. Ex vivo investigations confirmed an adhesion defect of isolated LAD I neutrophils on inflamed endothelial cells, indicating a defect in β_2_-integrin function [127]. Additionally, in in vivo assays performed with mice deficient for CD18, a LAD-I-related phenotype was detected [128].

LAD II is clinically characterized by mental retardation, periodontitis, and recurrent infections [129,130]. The rare disease is initiated by congenital disorders of glycosylation IIc (CDG-IIc) and dysfunction in fructose metabolism leading to immunodeficiency and psychomotor retardation. The glycosylation deficiency is based on the defective nucleotide sugar transporter, solute carrier family 35 member C1 (SLC35C1), transferring GDP-fucose into the Golgi apparatus [131]. In addition, LAD II leukocytes are characterized by a defect in sialyl Lewis x expression. Since PSGL-1 binding to E- and P-selectin on the inflamed endothelium is dependent on sLe^x^ expression, selectin–ligand binding and neutrophil rolling are impaired in LAD II patients [132]. Studies using LAD II leukocytes for ex vivo assays demonstrate a reduction of overall rolling events and an increased rolling velocity due to a failure of E- and P-selectin–ligand binding [127,133,134]. In vivo experiments with Slc35c1-deficient mice reveal a loss of rolling leukocytes in inflamed cremaster muscle venules, indicating an impaired selectin function resulting in a LAD II phenotype [135].

LAD III patients exhibit a mutation in the FERMT3 gene encoding kindlin-3, resulting in defective inside-out signaling and augmented integrin activation. Thus, leukocytes fail to arrest and migrate, causing an immune deficiency similar to LAD I. Patients suffering from LAD III mainly manifest a higher bleeding tendency due to a platelet aggregation deficiency, as well as recurrent infections [124,136]. CalDAG-GEFI is involved in β_1_- and β_2_-integrin activation within the leukocyte recruitment cascade. In CalDAG-GEFI-deficient mice, β_2_-integrin mediated adhesion is defective, resulting in an LAD III phenotype [137]. Further detailed information about the LAD diseases are described in excellent reviews and publications [4,138,139].

Many common chronic inflammatory diseases including atherosclerosis, diabetes, and periodontitis may be accompanied with late-onset Alzheimer’s disease [140]. The innate immune mechanism of the central nervous system is crucial in Alzheimer’s disease [141,142]. Neutrophils from Alzheimer patients are characterized by increased intravascular adhesion and intraparenchymal migration. Neutrophil extravasation into the brain results in extensive tissue damage. A study by Zenaro and colleagues reported an amyloid-β-induced LFA-1 activation, regulating neutrophil arrest and migration. Murine models of neutrophils lacking LFA-1 demonstrate a prevention from cognitive dysfunction and a reduction of gliosis. Inhibition of neutrophil trafficking via LFA-1 blockade may be useful in Alzheimer’s disease [143].

Thrombus formation and platelet–leukocyte aggregation may permanently obstruct blood flow, inducing organ dysfunction including tissue infarctions [144]. Platelets from patients suffering from thrombosis exhibit a strong increased P-selectin expression and subsequent binding to PSGL-1 on leukocytes [145,146]. Murine models using P-selectin- and PSGL-1-deficient mice demonstrated a reduction in platelet–leukocyte aggregates, indicating an important role of P-selectin and PSGL-1 in thrombosis formation [147]. Inhibition of these adhesion molecules may be a pharmacological target in antithrombotic therapy.

Sickle cell disease (SCD) is characterized by sickle red blood cells blocking small vessels and initiating pathological inflammation and thrombosis. In vivo studies demonstrate that P-selectin on activated platelets and endothelial cells binds PSGL-1 on neutrophils and unknown ligands on sickle cells in SCD [148]. These P-selectin-dependent neutrophil-platelet–erythrocyte aggregates were highly increased in SCD patients [149]. Recently, an in vivo study reported that P-selectin-deficient SCD mice are protected from lung vaso-occlusion, suggesting P-selectin inhibition as a useful clinical target in SCD [150]. However, another study using P-selectin-deficient mice in SCD demonstrated an increase in cellular liver senescence and a reduction in the epithelial cell proliferation [151]. Hence, the long-term effects of P-selectin inhibition in patients suffering from SCD have to be further investigated. Furthermore, in patients suffering from the coronavirus disease 2019 (COVID-19), thrombotic complications are common. Gene expression analysis of resting platelets of COVID-19 patients revealed significantly elevated P-selectin expression. Additionally, circulating platelet–leukocyte formation was faster and the number of aggregates was increased [152].

In conclusion, β_2_-integrins and selectins have been shown to play a crucial role in different human inflammatory diseases and are, therefore, potential targets for therapeutic approaches. Further investigations are necessary to completely understand their functions.
